# Phenotype as Agent for Epigenetic Inheritance

**DOI:** 10.3390/biology5030030

**Published:** 2016-07-08

**Authors:** John S. Torday, William B. Miller

**Affiliations:** 1 Harbor-UCLA Medical Center, Torrance, CA 90502, USA; 2 Independent Researcher, Paradise Valley, AZ 85253, USA; wbmiller1@cox.net

**Keywords:** phenotype, Darwin, Lamarck, germline, epigenetic, life cycle, niche construction

## Abstract

The conventional understanding of phenotype is as a derivative of descent with modification through Darwinian random mutation and natural selection. Recent research has revealed Lamarckian inheritance as a major transgenerational mechanism for environmental action on genomes whose extent is determined, in significant part, by germ line cells during meiosis and subsequent stages of embryological development. In consequence, the role of phenotype can productively be reconsidered. The possibility that phenotype is directed towards the effective acquisition of epigenetic marks in consistent reciprocation with the environment during the life cycle of an organism is explored. It is proposed that phenotype is an active agent in niche construction for the active acquisition of epigenetic marks as a dominant evolutionary mechanism rather than a consequence of Darwinian selection towards reproductive success. The reproductive phase of the life cycle can then be appraised as a robust framework in which epigenetic inheritance is entrained to affect growth and development in continued reciprocal responsiveness to environmental stresses. Furthermore, as first principles of physiology determine the limits of epigenetic inheritance, a coherent justification can thereby be provided for the obligate return of all multicellular eukaryotes to the unicellular state.

## 1. Introduction

The recognition that the cell is the basis for eukaryotic evolution [1] as a manifestation of perpetual cellular principles renders phenotypes as epiphenomena, i.e., subordinate to the actual event. Such a perspective alters many otherwise dogmatic aspects of evolutionary biology. In particular, the systematic error of the perception of evolution as a stochastic phenomenon yields instead to phenotypes as mechanistic products, always directed towards identifiable cellular needs [2]. Such a change of focus is similar in type to David Bohm’s insight into dual explicate and implicate orders in the physical realm [3]. He stipulated that our evolved senses mislead us into regarding our conscious experience as an explicate ordering of an entire reality. Instead, a truer reality is a continuous stream sustained by both explicates and an additional set of subjective implicates of which we are not typically aware. Similarly then, in biologic terms, it can be presented that an explicate phenotype is fully dependent upon a steady flow of epigenetic implicates in a cellular continuum that mechanistically interconnects evolutionary development with the larger environment across space and time.

## 2. Background

In conventional terms, any phenotype is assumed to be the end result of descent with modification through Darwinian random mutation and natural selection [4]. However, with the emergence of a contemporary re-appraisal of the importance of Lamarckian inheritance, the role of phenotype must be reconsidered as the effective primary means by which all organisms acquire information from the environment in the continuous maintenance of essential cellular requirements. These necessities are expressed through all the cellular mechanisms that are directed towards sustaining cellular activity within homeostatic limits, defending cellular integrity and self-recognition. Thus, in eukaryotic multicellular organisms, phenotype becomes an agent promoting and incorporating epigenetic inheritance, rather than a simple manifestation of the concordance of intergenerational vertical genetic transmission exclusively based on selection.

This perspective on the significance of the phenotype is consistent with Niche Construction Theory [5,6] critically enacted at the cellular level. When cellular imperatives or principles such as maintenance of preferential homeostatic status and self-protection in both individual and collective terms are subjected to environmental stresses through epigenetic inheritance, evolution is understood as much more dynamic and environmentally interactive than via any filtering mechanism of Darwinian evolution. Importantly, this perspective faithfully reflects evolution’s origin as a self-organizing, self-referential mechanism that originates within the unicellular domain, and always remains contingent on it [6]. In this manner, phenotype becomes a directed product of cellular activities in response to epiphenomena rather than a mere result of random forces [7].

Perhaps even more importantly, the impact of epigenetic inheritance on the cell, and its physiologic limits, is amenable to hypothesis testing and falsification in a manner beyond any generally accepted Darwinian evolutionary narrative [8]. Selection still applies, but its precise role and its center of action are deeply reconsidered.

## 3. The Water-Land Transition as the Epitome of Epigenetic Inheritance

There is evidence that life was initiated and then propelled on its evolutionary course in response to the physical constraints imposed by the environment [9], and therefore evolved in response to such major effectors as gravity [10], carbon dioxide [11], oxygen [12] and calcium [13] as epiphenomena to the cell [1].

The vertebrate transition from water to land was caused by the evaporation of water globally about 300 million years ago due to the accumulation of carbon dioxide in the atmosphere, causing a “greenhouse effect” [14]. An essential set of evolved traits was necessitated for the transition from water to land, critically dependent upon the lung as homologous with the swim bladder of bony fish [15]. This is particularly the case for physostomous fish [15], which have the homolog of a trachea (called the pneumatic duct) connecting the esophagus to the swim bladder. The swim bladder is derived from the foregut in both fish and land dwelling vertebrates [16]. Functionally, the effective inflation and deflation of both the swim bladder and lung are dependent on the production of surfactant by the gas gland epithelium lining the bladder lumen [17,18]. In the case of the swim bladder, the surfactant has been speculated to be necessary for preventing self-adherence of the walls of the bladder [19]. In the case of the lung, surfactant is necessary for preventing atelectasis, or alveolar collapse [20]. Alveoli are very small in diameter, thereby generating high surface tension based upon the Law of Laplace [21]. The physiologic stress of hypoxia forced selection pressure for the remodeling of the alveoli. The cell-cell interactions between the epithelial and mesenchymal components that mediate surfactant production [22] were modified through phylogeny and ontogeny in order to allow for the reduction in alveolar diameter, increasing the surface area-to-blood volume ratio for efficient gas exchange [23].

This mechanism for facilitated gas exchange, ever-dependent upon lipids, refers to the inception of cholesterol synthesis and its critical insertion into the cell membrane [24]. The facilitation of gas exchange through this cellular example of niche construction exemplifies how first principles of cellular requisites are put in service for oxygenation in unicellular organisms, exapted over billions of years through the implementation of homologous genetic motifs [25].

Starting from its origins, the spontaneous generation of micelles as lipids immersed in water [26], the reduction in entropy [27], in combination with chemiosmosis [28] and homeostasis [29] are assumed to have fostered life [30]. Unicellular life dominated the Earth for the first 4 billion years [31]. Then, fewer than 500 million years ago, multicellular organisms evolved from unicellular forms, likely due to competition among prokaryotes able to mimic multicellularity through traits such as Biofilm [32] and Quorum Sensing [33]. Rising levels of oxygen in the atmosphere put selection pressure on prokaryotes, producing hopanoids that caused increased order within the cell wall [34], making it more permeable. The generation of oxygen by bacteria was hypothesized to have given rise to cholesterol, given that eleven atoms of oxygen are required to form one molecule of cholesterol [35]. The presence of cholesterol in the cell membrane of primitive eukaryotes promoted metabolism, oxygenation and locomotion, the basic principles of vertebrate evolution [36]. There was also an increase in atmospheric carbon dioxide [37,38], which dissolved in the oceans to form carbonic acid [39]. That acidity leached calcium out of the bedrock, threatening life due to the denaturing effects of calcium on proteins, lipids and nucleic acids. In response, unicellular organisms formed peroxisomes, organelles that use lipids to buffer intracellular calcium [40]. In such a scenario, the formation of calcium channels from lipids for the excretion of calcium was exapted to protect burgeoning eukaryotes. During the Phanerozoic Era, the greenhouse effect of rising levels of carbon dioxide [13] forced some evolving vertebrates to transition from water to land [41,42,43], marking the beginnings of terrestrial life [44]. The adaptation to land gave rise to novel physiologic traits that had their origins in fish. The increased force of gravity on land [45] put great selection pressure on the skeletal system, altering it at least five times based on the fossil record [44]. Rising, fluctuating oxygen levels in the atmosphere necessitated remodeling of the internal organs, though there is no fossil evidence for these events. For example, we now know that the same genes that determine the swim bladder of bony fish determine the development of the lung [14]. Functionally, both the swim bladder and lung depend upon surfactant for their function [19,22], and the mechanisms that facilitated the evolution of the lung alveoli from the swim bladder delineate how and why these structures became smaller and more numerous [23] due to cellular interactions fostering evolutionary change. Moreover, the genes responsible for both skeletal and pulmonary evolution are involved in the evolution of the skin, kidney and brain. These adaptive changes were the net result of physiologic stress mediated by cellular-molecular damage to specific tissues and organs due to shear stress on microvessels generating radical oxygen species causing gene mutations and duplications [46]. This epigenetic remodeling pathway based upon the water-land transition exemplifies how phenotype becomes a product of environmental stress based upon cellular requisites in the cellular context of niche construction.

As apart from these deeply rooted cellular/molecular developmental mechanisms, there are now many direct examples of epigenetic factors influencing phenotype. The phenotypic differences between human monozygotic twins are now ascribed to epigenetic factors [47]. As an outgrowth of twin studies, there is a greater understanding of that link between epigenetics and phenotype. Such studies on genetically identical organisms suggest that studying the effects of epigenetics on phenotypic outcomes can yield discrete molecular pathways and mechanisms [48]. Therefore, an exploration of the effect of contemporary epigenetic impacts on phenotype can be expected to integrate with deep evolutionary experiences along the same types of molecular pathways that have been outlined above.

Certainly contemporary impacts can have substantial phenotypic results. In humans, maternal body mass index and blood pressure directly correlate with fetal birth weights [49]. Neonates born to obese women are larger and at a higher risk of birth complications. A similar association exists for elevated maternal fasting blood glucose, whereas elevated maternal systolic blood pressure has been directly linked to low birth weight infants. Starvation is now known to have profound intergenerational effects on phenotype, fitness and health in many animals. In *C. elegans*, generations of progeny of starved animals demonstrate smaller size, diminished fecundity, smaller brood size, a greater number of male progeny, and an increased tolerance to heat [50]. These transgenerational starvation effects in *C. elegans* have been demonstrated to be due to small RNAs that persist for at least three generations [51]. Such effects are now acknowledged in humans with starvation-induced neonatal adiposity and an increased incidence of diabetes in progeny [52]. Gluckman and Hanson [53] include the periconceptional, fetal, and infant environments among those aspects of particular significance in the future incidence of adult human disease. They further stress the dependence of the mature phenotype upon both any individual genome and its epigenome, which then, together and iteratively, influence subsequent responses to environmental stresses and disease incidence [54]. This emphasis on the reciprocating balance between the environment and phenotype via epigenetic intermediaries is a form of interrelating niche construction through which any particular organism receives feedback from the environment, is shaped by it in some manner, and then correspondingly affects the outward environment that it occupies.

Furthermore, the influence of epigenetic impacts on phenotype extends beyond those forms that are typically considered. The mammalian placenta represents an example of epigenetic interactions and their critical impact upon mammalian development that achieve complex phenotypic form. Mammalian placental development is partially dependent upon crucial reproductive protein expression that does not emanate from within any central genome. Instead, it is the product of early epigenetic impacts as a co-option of retroviral proteins. Such retro-elements are largely responsible for the formation of the placental syncytiotrophoblast [55]. The development of maternal immunosuppression enabling viviparity is itself critically dependent on proteins produced in response to accumulated retro elements as infectious epigenetic impacts on central genomes [56]. Furthermore, retrotransposon activity or suppression are now acknowledged as epigenetic mediators of phenotypic variation in mammals producing variations between genetically identical individuals [57]. Nor are such impacts of little consequence to genomic integrity since retrotransposons are the principal component of most eukaryotic genomes and alter the expression of a wide variety of genes in animals and plants [58]. Beyond these distant considerations, epigenetic impacts are actually now contemporaneously evident. The endogenization of HIV [59], Koala retrovirus [60], and the direct demonstration of heritable transmission of bacterial DNA [61] are crucial examples. Importantly, these transfers are emblematic of the self-same processes in which epiphenomena are either employed or withstood, within both the eukaryotic and prokaryotic realms [62]. Self-same processes is the descriptive term used to indicate that our evolutionary system is based upon cellular activities in which there are consistent adherencies to basic cellular principles. These basic mechanisms guide cellular interactions and reactions and are consistently reiterated at every scope and scale. In every circumstance, these physical and bioactive epiphenomena can now be appreciated as directing responses to environmental stress through reiterative means.

## 4. Predictive Value of Phenotype as Epigenetic Agent

Based upon these considerations, niche construction, either as beaver dams or cities, and then even further as phenotypes, can be productively assessed as consequences of elemental cellular first principles and epigenetic underpinnings. When considered in this manner, even our protracted human infancy and childhood can now be understood as a necessary phase through which crucial environmental epigenetic marks are assimilated to foster human brain development.

With these clarifications, all phases of the life cycle can be understood as derivative of cellular needs and imperatives that determine the timing and expression of each developmental and life cycle stage of which, arguably, the endocrine system has primacy. Crucially though, the endocrine system itself is a cellular phenomenon that is its own summation of epigenetic marks and their differential activation, ever-dependent upon environmental stresses [63].

## 5. Conclusions

In any typical Darwinian narrative, phenotype is an output of selection experienced through differential survival and reproductive success. However, heritable epiphenomena are now better understood. Therefore, it can be argued that epigenetic mechanisms are a primary means by which organisms evolve in matched reciprocation to environmental stresses best exemplified by niche construction. Phenotype can then be reappraised through a non-intuitive Bohmian shift [3] within biologic terms of co-existent implicates and explicates. Phenotype is a transient explicate upon which a series of epigenetic impacts gather, as a set of implicates, to be enacted according to cellular imperatives. The obligatory return of eukaryotic multicellular organisms to the unicellular form becomes the critical phase for the settling of those implicates towards biological expression as phenotype. Thus, a renewed evolutionary narrative can be considered that centers upon the primacy of epigenetic inheritance within deeply rooted cellular mechanisms. In such circumstances, perpetual cellular imperatives determine our evolutionary course. Phenotype is no longer merely a result but is instead a means through which organisms explore the outward environment and its stresses. Those impacts are brought back to the eukaryotic unicell and then, upon reproductive elaboration, enable the reiterative extension of phenotype into the environment to experience a subsequent series of environmental impacts towards its next set of adjustments. It is this consistent reciprocation that shapes phenotype. When fully considered, this new concept becomes a novel and testable route towards further progress in evolutionary theory and biomedicine.
